# Chromosome structural variation in tumorigenesis: mechanisms of formation and carcinogenesis

**DOI:** 10.1186/s13072-020-00371-7

**Published:** 2020-11-10

**Authors:** Wen-Jun Wang, Ling-Yu Li, Jiu-Wei Cui

**Affiliations:** Cancer Center, The First Hospital of Jilin University, Jilin University, Changchun, 130021 Jilin China

**Keywords:** Structural variation, Cancer, Translocation, Chromothripsis

## Abstract

With the rapid development of next-generation sequencing technology, chromosome structural variation has gradually gained increased clinical significance in tumorigenesis. However, the molecular mechanism(s) underlying this structural variation remain poorly understood. A search of the literature shows that a three-dimensional chromatin state plays a vital role in inducing structural variation and in the gene expression profiles in tumorigenesis. Structural variants may result in changes in copy number or deletions of coding sequences, as well as the perturbation of structural chromatin features, especially topological domains, and disruption of interactions between genes and their regulatory elements. This review focuses recent work aiming at elucidating how structural variations develop and misregulate oncogenes and tumor suppressors, to provide general insights into tumor formation mechanisms and to provide potential targets for future anticancer therapies.

## Introduction

Widespread chromosomal genomic rearrangement and point mutations are underlying hallmarks of the cancer genome. Next-generation sequencing technology has enabled the detection of diverse patterns of genomic changes in human somatic cells. Chromosome structural variation, a vital kind of somatic mutation, is involved in the process of genomic rearrangement ranging from genes to entire chromosomes, and also affects gene expression regulation. Chromosome structural variation is a vital driver of oncogenesis and progression in both solid tumors and hematopoietic malignancies [1]. The combination of clinical features and structural variations provides the opportunity for cancer diagnosis, reasonable tumor subtype classification, prognosis, and precision treatment [2]. In fact, clinical testing for specific mutations and genomic classification has achieved overwhelming successes in hematology [2]. With the development of molecular biology techniques, many specific chromosomal structural variations (e.g., *TMPRSS2-ERG* and *EML4-ALK*) have also been identified in solid tumors in recent years.

The Pan-Cancer Analysis of Whole Genomes (PCAWG) Consortium of the International Cancer Genome Consortium (ICGC) and The Cancer Genome Atlas (TCGA) collaborated to analyze structural variants, genomic breakpoint cluster regions, and gene expression based on whole-genome sequencing data from 2658 cancers patients across 38 tumor types [3, 4]. However, our understanding of the underlying molecular mechanisms of structural variation remains incomplete. Nonetheless, accumulating evidence suggests that changes in the three-dimensional (3D) conformational composition or topologically associated domains (TADs) provide a viable explanation for aberrant gene expression in structural variation [5]. Moreover, the higher-order chromatin structure as well as epigenetic modifications (especially modifications to histones and DNA) has gradually received interest as their vital roles in genome instability and DNA repair [6–9]. A greater understanding of the specific molecular mechanisms involved in the process of chromosomal rearrangement will provide invaluable guidance for the design of precise cancer therapies. Because driver mutations are causative, it is therapeutic to target the function of resulting proteins or decrease the occurrence of structural variation in tumors.

This review builds on our increasingly sophisticated understanding of structural variation due to scientific advances made over the last decade. Our goal is to summarize the mechanisms of structural variations in tumorigenesis from both molecular mechanisms and spatial structure, to convey a novel perspective for clinical therapies and resistance prevention.

## Categorization of structural variants

Although the patterns of structural variants are different, their formation is commonly involved in the occurrence of DNA double-strand breaks (DSB) and improper repair or rejoining of broken chromosomes [10]. The number of breakpoints involved and the rearrangement patterns are two significant features of structural variants. Li et al. have suggested that these structural variants should be categorized according to the two aforementioned factors [1]. In terms of the number of breakpoints, structural variants can be divided into simple (i.e., deletions, tandem duplications, reciprocal inversions, and translocations) or complex structural variants (i.e., chromothripsis and chromoplexy among others). Inversions can be further divided into paracentric and pericentric inversions. Deletions are the most common and simplest structural variant, followed by tandem duplications and unbalanced translocations [1].

Meanwhile, the rearrangement patterns include ‘cut-and-paste’ (e.g., deletions, reciprocal inversions, chromothripsis, and chromoplexy) or ‘copy-and-paste’ (e.g., tandem duplications, templated insertions, and local *n*-jump) [1]. The templated insertions refer to a string of inserted segments copied from one or more genomic templates. The local *n*-jump is a cluster of n structural variations located at a single genomic region accompanied by copy number alterations (CNAs) as well as inverted and noninverted junctions. Breakage-fusion-bridge events are more complex ‘cut-and-paste’ processes that produce structural variants and are caused by cycles of DNA breakage, end-to-end sister chromatid fusions, mitotic bridges, and further DNA breakage. Such events can result in non-reciprocal translocations, ‘fold-back inversions’, and loss of heterozygosity [11].

## Formation of structural variants

Accurate inference of specific structural variants is crucial for further characterization of the underlying molecular process. It is quite difficult to recognize large-scale complex chromosome rearrangements, which often affect multiple genes, simultaneously. The constant development of novel sequencing technologies and bioinformatic tools provides opportunities to explore the associated epidemiology and mechanisms of rearrangements. Several mechanisms have been thought to give rise to genomic rearrangements involving DNA breakage, improper DSB repair, and long interspersed element-1 (LINE-1 or L1)-mediated retrotransposition.

### DNA breakage

#### Simple DNA breakage

The patterns of chromosome rearrangement are influenced by the cause and position of DSBs as well as the specific mechanisms involved in the repair. DSBs are the most lethal type of DNA damage and are important intermediates in the process of generating structural variants. Exogenous mutagens (e.g., chemicals, ionizing radiation, ultraviolet light, and viral infections) or cell-intrinsic processes (e.g., oxidative metabolism or replication stress) that occur throughout life often produce somatic mutations [2, 12, 13]. Endogenous DSBs mainly occur during fundamental processes, especially replication and transcription (reviewed by Cannan et al. and Bouwman et al.) [14, 15]

Activation-induced cytidine deaminase (AID) and recombination-activating genes (RAGs) can both generate off-target DNA cleavage, thereby contributing to genomic rearrangements in cancer [16, 17]. AID accounts for genetic changes (including somatic hypermutation and class-switch recombination [CSR]) in the Ig gene in activated B cells. Interestingly, however, AID can target non-Ig genes, leading to DNA DSBs or mutations [17]. RAG, a lymphoid-specific endonuclease, participates in V(D)J recombination, which accounts for antigen receptor diversity [16, 18]. Type II topoisomerase (TOP2) proteins, which normally solve topological issues through DSBs, can generate several known translocation breakpoints in leukemia and prostate cancer (PCa) resulting in *MLL* and *TMPRSS2-ERG* translocations, respectively [19, 20].

Telomere dysfunction can also cause genome instability which may trigger oncogenic events [11]. During DNA replication, chromosomes with uncapped telomeres are recognized as DNA double-strand ‘breaks’ resulting in the generation of end-to-end fusions and subsequently, a dicentric chromosome. The spindles can attach to both centromeres, leading to the dicentric chromosome being pulled in opposite directions during mitosis, thus giving rise to breaks at random positions [11]. The resolution of chromatin bridges between daughter cells by nucleases in telophase can also result in stretches of single-stranded DNA, which can serve as substrates for cytidine deaminases such as apolipoprotein B mRNA editing enzyme, catalytic polypeptide-like 3B (APOBEC3B), which predominately mediates C → T mutations. PCAWG analysis showed that kataegis (i.e., clusters of localized hypermutation) was described in 60.5% of all cancers, is associated with somatic structural variant breakpoints, and contributes significantly to tumor heterogeneity [4].

DNA sequence features and chromatin properties play a vital role in the breakage susceptibility of genome regions, occurrence, and non-homogeneous distribution of DSBs in the cell nucleus [21–23]. In leukemia, some genomic breakpoints tend to cluster in certain intronic regions of the relevant genes, rather than being distributed throughout the whole gene [24]. Compared with compact chromatin, open chromatin containing active genes is more prone to radiation damage [21, 23]. Genome-wide analyses and ChIP-seq data have shown that transcriptionally active loci, protein binding sites, or transcription start sites (TSS) are particularly sensitive to breakage [25, 26]. For example, in PCa with the *TMPRSS2-ERG* rearrangement, rearrangement breakpoints were enriched with open chromatin marks such as H3K4me3, H3K36me3, and H3ace [23]. Several genes (such as *MALAT1 and SNHG3*) which are targeted by AID are involved in translocations in tumors and their break sites are enriched for H3K4me3 and repetitive sequences [17].

#### Complex DNA breakage

Chromothripsis, which was identified in 2011, refers to massive genomic rearrangements that result from a single catastrophic event and are clustered in isolated chromosomal regions during early tumor evolution [27, 28]. This phenomenon provides a mechanism that allows for rapid accumulation of hundreds of rearrangements during a single event, contrary to the traditional concept of malignant transformation. Chromothripsis can be classified as classical or balanced chromothripsis. The loss of DNA fragments due to a catastrophic chromosomal shattering can lead to copy number oscillations between two or three states and interspersed loss of heterozygosity (LOH) in the fragmented chromatid, which has a single copy of the parental homolog. At the same time, balanced chromothripsis involves a smaller number of rearrangement breakpoints compared to classical chromothripsis, and most of the DNA fragments are preserved and reassembled after shattering of the DNA [29]. Surprisingly, chromothripsis generally has a prevalence of up to 29% of high-confidence calls (which refer to oscillations between two states in > 7 adjacent segments) as well as 40% of low-confidence calls (which refer to oscillations in 4–6 segments). Interestingly, chromothripsis is frequently found in liposarcomas (100%) and osteosarcomas (77%) [27].

Until now, various cellular processes have been proposed to explain chromothripsis, such as micronuclei formation, telomere dysfunction, mitotic errors, abortion of apoptosis, premature chromosome compaction, and ionizing radiation acting on condensed chromosomes [30]. Chromothripsis involves only a single chromosome or part of a chromosome, thereby suggesting that the affected chromosome may undergo a period of spatial isolation from the remaining genome. Therefore, the most favored hypothesis to explain chromothripsis activation is micronuclei formation due to chromosomal mitotic physical isolation failure [31]. The chromosome(s) lagging in anaphase are encapsulated into the micronuclei, which can be missegregated and excluded from the main nucleus upon cytokinesis. Replication stress and DNA damage repair are in synergy to further contribute to DSB formation and micronucleus-associated chromothripsis (reviewed by Guo et al.) [32]. Second, as mentioned above, another potential mechanism is the attack on chromatin bridges by nucleases, which can also shear the DNA into hundreds of pieces in cells with telomere dysfunction in the breakage–fusion–bridge cycle [28]. These pieces can also give rise to micronuclei in one or both daughter cells at the end of mitosis [32]. Third, premature chromosome compaction is an event that occurs in chromosomes before DNA replication can be completed, consequently resulting in the shattering of incompletely replicated chromosomes [30]. Moreover, the abortion of apoptosis and hyperploidization are thought to be causes of chromothripsis. However, based on recent PCAWG analyses, *p53* inactivation and polyploidy are predisposing factors, but not prerequisites for chromothripsis, since 60% of the chromothripsis cases do not contain *TP53* mutations [27].

Apart from complex rearrangements that occur in isolation resulting in shattered DNA, such rearrangements can also involve one, two, or more chromosomes (i.e., chromoplexy). Chromoplexy, another pattern of complex structural variation identified in PCa genomes in 2013, has many interdependent structural variant breakpoints (mostly interchromosomal translocations) [33]. Chromoplexy is prominent in prostate adenocarcinomas, lymphoid malignancies, and thyroid adenocarcinomas [4]. The micronucleus-based model can also mediate the process of chromoplexy if multiple chromosomes are packaged in micronuclei [32].

#### Roles of epigenetics and 3D genome organization in DNA breakage

The higher-order chromatin structure or chromatin status can remarkably influence the susceptibility of the DNA to damage. The human genome is three-dimensionally organized into TADs that usually maintain a high level of conservation; however, these are often disrupted in several diseases (e.g., cancers). CCCTC-binding factor (CTCF), a highly conserved nuclear phosphoprotein frequently present at TAD boundaries, plays a causal role in the formation and maintenance of TADs and loops through direct interaction with cohesin [34–36] (Fig. 3a). As an ATP-driven molecular machine, Cohesin-NIPBL extrudes DNA loops bidirectionally through nontopological interactions with DNA until two CTCF-binding sites are found in convergent orientation [37, 38]. In contrast, chronic depletion of CTCF dysregulates steady-state gene expression by subtly destroying TAD boundary integrity and altering transcriptional regulation, which can also be observed in primary tumors [36, 39]. Chromatin loop anchors points or TAD boundaries are considered as hotspots for DNA breakpoints and rearrangements [6, 7]. In leukemias, the TOP2-induced DSBs are reported to accumulate at those genes which have high transcriptional activity and are enriched at chromatin loop anchors [19]. Common fragile sites refer to large genomic regions with active transcription and high rates of DNA breakage under replication stress [40]. A recent study demonstrated that common fragile sites span TAD boundaries and harbor highly transcribed large genes (> 300 kb) with sensitivity to replication stress caused by DNA polymerase inhibitor aphidicolin [40]. Mechanically, the selective pressure to maintain intact TADs and molecular property of the chromatin at TAD boundaries jointly result in this phenomenon [7]. TAD boundaries are locally transcriptionally active and open chromatin structure with enrichment in H3K4me3, CTCF, CpG islands, and SINE elements [41].

Besides, in V(D)J recombination and CSR, cohesin-mediated chromatin loop extrusion has been demonstrated to mediate the juxtaposition of translocation loci and can lead to pathogenic chromosomal translocations [42, 43]. In PCa, androgen receptor (AR) can bind intronic binding sites near the tumor translocation sites and further promote spatial proximity in a ligand-dependent way [44]. Intronic AR binding can also cause accumulation of dimethylation of histone H3 lysine 79 (H3K79me2) and H4K16 acetylation at DSB sites, thereby conveying sensitization to genotoxic stress [44]. Meanwhile, AID and LINE-1 repeat-encoded ORF2 endonuclease are recruited by AR to generate DSB in these specific regions, thus resulting in specific chromosomal translocations of PCa [44].

On the other hand, chromatin architecture can be regulated by epigenetic modifications, such as acetylation, methylation, phosphorylation, and ubiquitination. H3K27me3 and H3K9me2/H3K9me3 are characteristics of heterochromatin, which is less sensitive to DNA damage [45]. In contrast, the protein binding and open chromatin are found to enrich in the vicinity of DSB sites [26]. In PCa, chromoplexy breakpoints tend to have a connection with active transcription and open chromatin configurations and cluster in actively transcribed DNA with high GC content [33]. H3K79me has been found at active loci for V(D)J recombination in B cells [46]. TADs contain various epigenetic marks and influence expression levels of many genes within the TAD [47]. Manipulating epigenetic modifiers can regulate the chromatin architecture and may represent a potential way to raise cancer cell sensitivity to cancer therapies such as gamma-rays.

### Two-ended DSBs repair-based rearrangement mechanisms

**Fig. 1:**
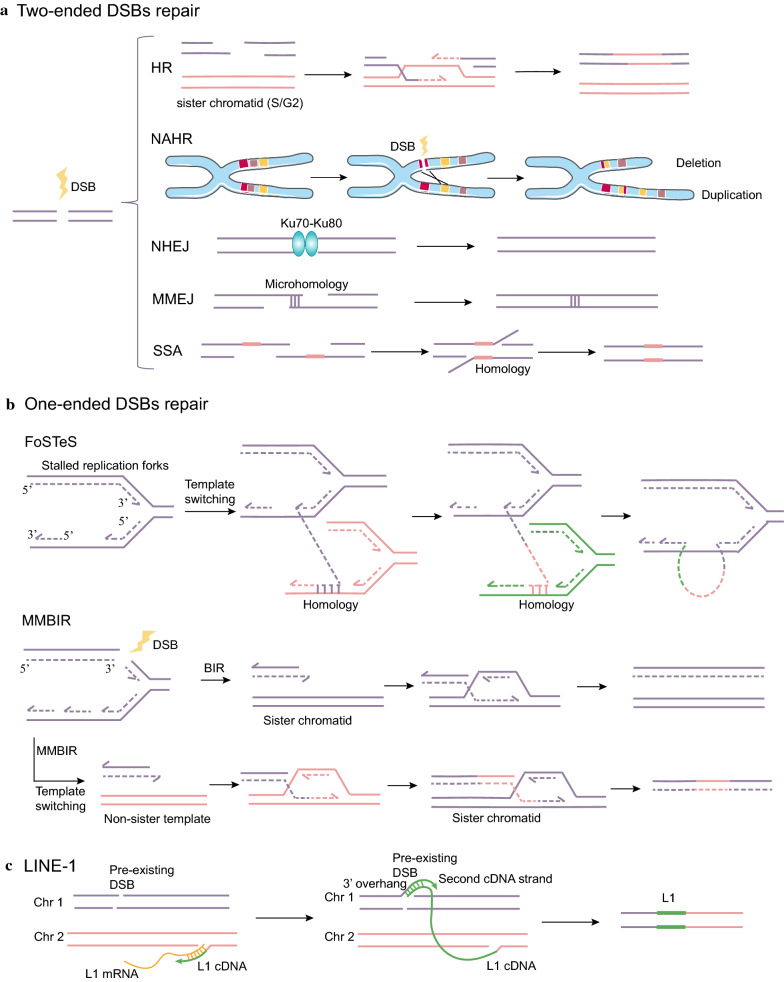
Proposed mechanisms involved in the formation of structural variation.** a** DSBs can be repaired by HR, NAHR, NHEJ, MMEJ, and SSA. **b** FoSTeS and MMBIR model. During DNA replication, the DNA replication fork can stall, leaving the lagging strand to invade another replication fork using complementary template microhomology to anneal and extend by DNA synthesis. The failure to repair by BIR can induce MMBIR, which drives the strand invasion of non-sister templates using microhomology-containing regions, thereby giving rise to chromosomal rearrangements. **c** LINE-1 or L1-mediated retrotransposition. L1 retrotransposition can mediate the first-strand nick by the endonuclease, followed by first-strand cDNA synthesis with L1 mRNA as the template by reverse transcriptase. The cDNA negative-strand can invade a second 3′ overhang from a preexisting DSB and mediate the synthesis of the second-strand cDNA

Under normal conditions, two-ended DSBs are repaired either by homologous recombination (HR) or by non-homologous end-joining (NHEJ). In structural variants, different mechanisms for repairing broken chromosomes include HR, non-allelic homologous recombination (NAHR), NHEJ, microhomology-mediated end-joining (MMEJ, also known as alternative non-homologous end-joining [Alt-NHEJ]), and single-strand annealing (SSA; Fig. 1a). PCAWG data show a few overlapping sequences at most breakpoints in tumor genomes. Some of these breakpoints have microhomology (2–7 bp or 10–30 bp), suggesting that NHEJ is the most dominant DNA repair pathway, followed by MMEJ and SSA [1]. In chromothripsis, NHEJ is the most dominant DNA repair mechanism with partial contributions from microhomology-mediated break-induced replication (MMBIR) or MMEJ [30, 48, 49]. DNA damage can be sensed by several DNA damage sensor proteins (e.g., the MRE11/RAD50/NBS1 [MRN] complex and the Ku70–Ku80 heterodimer), which then recruit signal transducers (e.g., ataxia telangiectasia mutated [ATM] and CHK1) to further amplify the signal [50, 51]. Finally, these signaling cascades activate effectors (e.g., cell cycle regulators, DNA repair factors, apoptotic machinery, and chromatin modifications, among others) [50].

#### HR and NAHR

HR, a highly accurate repair mechanism, is based on a sister chromatid in the late S/G2 phase. In HR, poly(ADP-ribose) polymerase 1 (PARP1) recognizes the DSB and induces the relaxation of chromatin through recruitment of the chromatin remodeler Alc1. The MRN complex can bind the DSB, resulting in activation of kinase ATM and subsequent phosphorylation of ATM (pATM) and histone H2AX at serine 139 (γH2AX) [50, 52]. Subsequently, γH2AX can further recruit additional pATM and DNA damage response (DDR) proteins such as the p53 binding protein 1 (53BP1) [50]. In contrast, NAHR may reflect errors that occur during HR of DSBs or during directed recombination in meiosis (i.e., when the sister chromatid is absent) [53]. When a DSB occurs, homology search may instead find a nearby paralog of the repeat, leading to NAHR and subsequent rearrangement, including deletion, duplication, inversion, and translocation [54, 55]. NAHR events are enriched in compartments A or open chromatin [53]. The specific type of rearrangement lies in the physical proximity between homologous repeats, the location, and the orientation of the paralog with respect to the DSB [55].

#### NHEJ

NHEJ repairs DSBs by mediating the quick and direct religation of the broken ends without the need for a homologous template in any phase of the cell cycle. In NHEJ, DSBs are sensed by the Ku70–Ku80 (also known as XRCC6–XRCC5) heterodimer, which activates the protein kinase DNA-PKcs by protein–protein interactions, thereby contributing to the recruitment of end-processing enzymes, polymerases, and DNA ligase IV to the DNA ends [51]. Ku70–Ku80 tends to bind to either flush ends or short single-stranded DNA (ssDNA) overhangs, rather than long ssDNA overhangs [56].

#### MMEJ and SSA

In MMEJ, end resection by the MRE nuclease reveals microhomologies on 3′ ssDNA, which then anneal to guide repair. Moreover, SSA uses extensive end resection to reveal homologous repeat 3′ ssDNA ends, which can bridge DSB ends by annealing [57]. Next, the single-stranded tails are digested away, resulting in deletion between the repeats; therefore, SSA is thought to be an error-prone pathway.

#### Factors affecting DSB repair

The response of cancer cells to DSBs is different from that of normal cells because of a multitude of reasons, including the loss of cell cycle checkpoints and defects in the DSB repair system, resulting in unwanted chromosome rearrangements [58, 59]. Chromosome instability and rearrangements are associated with inherited and acquired defects in DNA repair genes, which are key mechanisms in the genesis of malignant tumors [48, 60]. Germline mutations in the transcription factor *HOXB13* and DNA damage repair genes (e.g., *BRCA1, BRCA2, ATM, CHEK2,* and *PALB2*) as well as mismatch repair (MMR) genes (e.g., *MSH6* and *PMS2*), have been proven to increase the risk of PCa [60]. In a *p53*-deficient background, inactivation of essential DNA repair factors in HR (e.g., *BRCA2*) or canonical NHEJ (e.g., *XRCC4* and *Lig4*) account for frequent complex genomic rearrangements in murine medulloblastomas or high-grade gliomas [48].

DSB signaling and repair are connected with their initial genomic localization and chromatin structure [21, 25]. Development of structural variations is now recognized as a nonrandom process since both the spatial genome organization and genome rearrangements are tissue- and cell-type specific [61]. Whole chromosomes within the nucleus often occupy distinct positions instead of being randomly distributed and are further organized into chromatin domains within different nuclear compartments [61, 62]. Most gene-rich chromosomes are localized in the central part of the nucleus, whereas the gene-poor chromosomes occupy more peripheral positions close to the nuclear membrane [21]. In fact, cancer-relevant translocations occur more frequently in chromosomes that are in spatial proximity or pre-positioned proximal DSBs, suggesting that such nonrandom positioning of chromosomes and genes may strongly contribute to specific initial oncogenic rearrangements in a given cell type or tissue [63–65].

Furthermore, genomic distance is a major determinant of interaction frequency [66]. Evidence from a study in budding yeast showed that the efficiency of HR is regulated by its proximity to the homologous locus [25, 67]. In interactions throughout the genome, intra-chromosomal interactions are much more frequent than inter-chromosomal interactions; at the same time, the interaction frequency decreases as the genomic distance increases within a single chromosome [62]. In the “contact-first” model, which is based on the 3D structure, spatial proximity has been shown to affect the likelihood of two DNA ends joining and partner selection for chromosomal structural variation [68]. In normal human cells, many chromatin domains that contain translocation partners are spatially proximal with elevated Hi-C contact frequencies, thus predisposing them to chromosomal rearrangements, such as *BCR-ABL* in chronic myeloid leukemia and *MYC-IGH* in Burkitt's lymphoma [68]. It should be noted that aggregate Hi-C signal from bulk/ensemble Hi-C analysis may not reflect the spatial interaction of rare cells population.

Moreover, the mobility of chromosome breaks also affects DSB repair and the probability of structural variation. Chromosome regions at the nucleoli or the nuclear periphery have been reported to be remarkably immobile in mammalian cells [69]. The mobility of DSBs is higher than that of intact chromatin to facilitate HR [70, 71]. There is no denying that the occurrence of a subset of translocations results from distally positioned DSBs that undergo long-range motion [64]. Indeed, many translocations occur in regions with low or average Hi-C contact frequency, suggesting that spatial proximity is certainly not a sufficient condition for translocation in cancer genomes [68].

#### Roles of ncRNAs in DSB repair

**Fig. 2:**
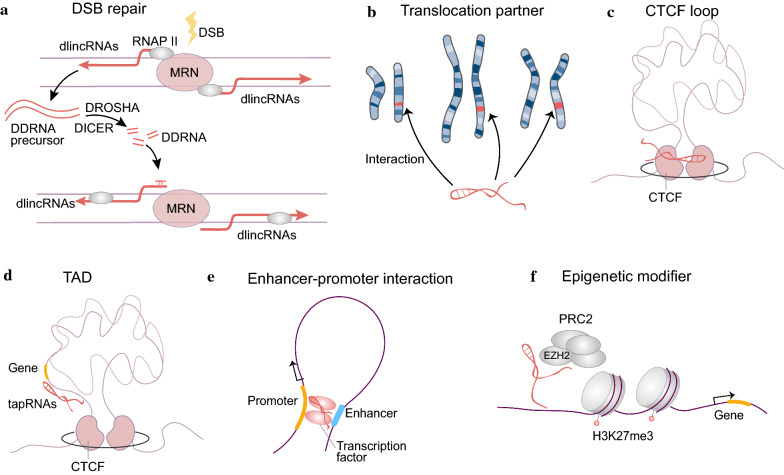
Roles of non-coding RNAs in structural variation.** a** dlincRNA-mediated DSB repair. After DSBs, RNA polymerase II (RNAPII) binds to the MRN complex and generates dilncRNAs, which are DDRNA precursors. Then, DDRNA pairs with nascent unprocessed single-stranded dilncRNAs, and they cooperate to bind to 53BP1 and fuel DNA damage response activation. **b** LncRNA can interact with multiple translocation partners. **c** RNAs are involved in the formation of chromatin loops through proper interaction with the RNA-binding region in CTCF. **d** The tapRNAs are co-expressed with their neighboring genes in a tissue-specific manner and they regulate genes by affecting topological conformations. **e** lncRNAs can modulate their target gene expression by promoting enhancer–promoter interactions. **f** RNAs interact with and recruit epigenome regulators such as components of PRC2 including EZH2 to the targeted locus, and then promote trimethylation of H3K27 in the targeted locus, thus inducing silencing of specific genes

Noncoding RNAs (ncRNAs), referring to RNAs that do not encode proteins, play key roles in DSB repair, maintenance of genome stability, organization of the 3D genome architecture, and control of gene expression through epigenetic mechanisms [72, 73] (Fig. 2).

Small RNAs generated at DNA break sites participate in DSB repair. Small ncRNAs, termed DNA-damage RNAs (DDRNAs), carrying the sequence of the DNA flanking the DSB, are generated at DSBs and are critical for DNA damage response activation [74]. Following DSBs, RNA polymerase II (RNAPII) binds to the MRN complex and generates damage-induced long ncRNAs (dilncRNAs), which are DDRNA precursors. Subsequently, DDRNA pairs with nascent unprocessed single-stranded dilncRNAs to bind to 53BP1 and fuel DDR activation [74, 75]. A recent study demonstrated that dilncRNAs can stimulate liquid–liquid phase separation of DDR factors (e.g., 53BP1), thereby driving the crowding of DDR proteins and promoting DDR foci formation and DSB repair [75, 76]. Long ncRNAs (lncRNAs) can also serve as scaffolds through interactions with several DNA repair proteins including, but not limited to, Ku70/Ku80 and 53BP1. LncRNA LINP1, which is upregulated in triple negative breast cancer, can be recruited to the DSB position by the Ku80–Ku70 heterodimer to provide a scaffold for Ku80 and the DNA-PKcs complex, thereby enhancing NHEJ activity [77].

Moreover, ncRNAs play roles in mediating DNA–DNA or DNA–protein interactions and in providing a repair scaffold. In acute myeloid leukemia (AML) with t(8,21), lncRNA RUNX1 overlapping RNA (RUNXOR) orchestrates an intrachromosomal interaction between translocation breaking sites and multiple *RUNX1* translocation partners [78]. These data suggest that lncRNAs may function as previously undefined chromatin factors that are involved in translocation by promoting spatial proximity.

#### Roles of epigenetic regulators and 3D chromatin topology in DSB repair

3D nuclear architecture and some epigenetic regulators also play a role in DSB repair. Several studies have established that TADs are instrumental for DDR foci formation and constraint of the DSB repair signaling. For example, through cohesin, the chromatin architecture regulates the spread of γH2AX from DSBs to TAD boundaries [50]. After DNA breakage, 53BP1 accumulate and assemble into 4–7 53BP1 nanodomains (each of which corresponds to a single TAD) and further form higher-order 53BP1 microdomains around DSB [79]. Then RIF1 and cohesin are speculated to be recruited to the boundaries of nanodomains to stabilize several neighboring TADs into an ordered, circular arrangement [79, 80]. Such ordered and stabilized 3D chromatin topology around DSB can protect DNA ends from excessive resection of enzymes and elevate the local concentrations of limiting anti-resection factors (e.g., shieldin), thereby safeguarding genome integrity [79]. Additionally, CTCF can be rapidly recruited to DSBs through zinc finger domain and serve as a scaffold for repair proteins [81]. Additionally, in response to DNA damage, the spatial positioning of some gene-rich chromosome territories alter, which is hypothesized to promote cell cycle arrest and increase the accessibility of DSB sites to repair proteins [82, 83].

Moreover, in DDR, chromatin status and histone modifications (e.g., ubiquitylation, SUMO, methylation, phosphorylation) also play a vital role in driving DNA damage signaling and recruiting repair proteins [8]. ATM is considered as the core of local chromatin alterations for accessibility to DSB repair events through histones post-translational modifications, reorganization of specific chromatin chromosomal domains, etc. [84]. The γH2AX domains in the vicinity of DSBs enable the binding of ATM-modified cohesion, which can regulate chromatin architecture and raise sister chromatid cohesion [85, 86]. Accumulation of γH2AX is speculated to modify charges within the chromatin domain and thus contributing to phase separation, which mainly relies on electrostatic interactions [87].

First, acetylation of histone lysines is a hallmark of a more relaxed chromatin state to facilitate DNA repair. In response to DSB, the histone acetyltransferase TIP60 and histone acetyltransferase (HAT) cofactor TRRAP can acetylate histone H4, thus inducing a more relaxed chromatin state and enabling access to repair proteins at DSB sites [88]. Second, histone methyltransferases such as Enhancer of Zeste Homologue 2 (EZH2) are recruited to the DSB site and induce H3K9me and H3K27me, thereby inhibiting transcription to fuel DNA repair [43]. Histone deacetylase 1 (HDAC1) and HDAC2 may remove part of the H3K27ac marks at DSB sites, thus contributing to the enrichment of EZH2-mediated H3K27me3 [9]. Third, RNF8/RNF168 and the polycomb repressive complex 1 (PRC1) are two identified histone H2A/H2AX/H2AZ-E3 ubiquitin ligases that initiate a series of ubiquitylation at DSB sites [89]. RNF8 is first recruited to the DSB sites in an ATM-dependent fashion and then cooperates with the E2 ubiquitin-conjugating enzyme, UBC13 to mediate K63 ubiquitylation of histone H1 [89, 90]. The ubiquitylated H1 can further recruit RNF168, which can propagate H2A ubiquitylation and induce recruitment of repair factors such as 53BP1 and BRCA1 [90]. Recently, RNF8 is reported to promote ALT-EJ and HDR [91]. Furthermore, ATM phosphorylates transcriptional elongation factor ENL, followed by recruitment of PRC1 [92] PRC1 contains the enzymatic subunit RING1A/B and polycomb group RING finger protein 4 (PCGF4, also known as BMI-1), which can mediate the ubiquitylation of γH2AX and thus switching off transcription [91, 93].

### One-ended DSB repair-based rearrangement mechanisms

Apart from two-ended DSBs, the DSB repair system still needs to cope with one-ended breaks resulting from broken replication forks, in which immediate end-joining partners are absent. This case involves the invasion of the 3′ ssDNA end to the donor chromosome (homologous or heterologous chromosome) and subsequent replication. Then, the separated end can dissociate and further reinvade another DNA template to iterate this process. A few rounds of microhomology-mediated template switching can account for complex breakpoints with multiple short sequences derived from different loci in some structural variants (such as local *n*-jump and templated insertion events) (Fig. 1b) [94]. There are two mechanisms, fork stalling and template switching (FoSTeS) or MMBIR.

#### FoSTeS

In the FoSTeS model, during DNA replication, the DNA replication fork can stall leaving the lagging strand to invade another replication fork using complementary template microhomology to anneal and extend by limited DNA synthesis [94]. Disengagement, invasion, and synthesis can continuously occur several times. The forks involved may be in proximity to chromosomal 3D space, but not necessarily adjacent to the original replication fork [94].

#### MMBIR

The classic break-induced replication (BIR) is a DSB repair model of replication restarting at broken forks based on the HR mechanism [95]. The failure to repair by BIR can induce MMBIR, which drives the RAD51-dependent strand invasion of non-sister templates using microhomology-containing regions, giving rise to chromosomal rearrangements.

### Transposable element-mediated retrotransposition

LINE-1 or L1 is the most active autonomous transposable element encoding endonuclease and reverse transcriptase (reviewed by Belancio et al.). Moreover, L1 exists in approximately 50% of human tumors [96]. L1 retrotransposition can mediate a first-strand nick by the endonuclease, followed by first-strand negative-strand cDNA synthesis with L1 mRNA as the template by reverse transcriptase. The cDNA negative-strand can invade a second 3′ overhang from a preexisting DSB and mediate the synthesis of the second-strand cDNA, leading to deletions, duplications, inversions, translocations, and breakage–fusion–bridge cycles [96, 97] (Fig. 1c).

## Carcinogenic mechanism of structural variation

Altered genes caused by structural variation may involve driver or passenger mutations through multiple mechanisms in tumorigenesis. Structural variations have functional consequences in tumorigenesis or clonal evolution through multiple mechanisms, such as CNAs, fusion gene rearrangements, and gene expression patterns (epigenetic alterations or inappropriate communication between genes and distal regulatory elements) [3, 5, 98]. Interestingly, the majority of genes with altered expression due to corresponding breakpoint events show upregulated gene expression [3]. Overall, although the vast majority of driver mutations occur in a protein-coding content, which makes up only 1% of the human genome, non-coding structural variations may be underappreciated mutational drivers in cancer genomes [99].

### Gene truncation and inactivation of genes

Conjoint analysis of structural variation and mRNA expression levels has been used to predict gene truncation affected by structural variation. In some tumor suppressor genes, including, but not limited to, *PTEN, TP53*, *RB1, NOTCH1,* and *NF1*, sequences or promoters can be frequently interrupted by translocation and inversion, leading to inactivation and the subsequent onset and progression of cancer [3, 100]. In addition, gene truncation can also result from templated insertions or local *n*-jumps, which cause duplications of internal exons of this gene or insertions of exons from other genes, leading to a nonfunctional transcript [1]. Apart from the tumor suppressor genes, the inactivation mechanisms of some chromatin modification genes (e.g., *DNMT3A*, *IDH1,* and *NSD1*) and DNA repair genes also involve truncation mutations in cancer [101].

### CNAs accompanied with corresponding alterations in gene expression

In cancer genomes, CNAs may frequently affect regulatory elements and genes linked to these regulatory elements in a dose-dependent manner, potentially contributing to oncogenesis [99]. CNAs, which are caused by duplications, deletions, or templated insertions, mainly affect coding genes, especially oncogenes or tumor suppressor genes, and are considered to drive mutation events in several types of cancers [102]. In 36% of clear cell renal cell carcinoma patients, simultaneous chromosome 3p (encompassing four tumor suppressor genes: *VHL, PBRM1, SETD2,* and *BAP1*) loss and 5q gain mostly results from chromothripsis [103]. Most amplifications are due to tandem duplications [104]. Data show that 81% of metastatic castration-resistant PCa patients have amplification of an intergenic enhancer region upstream of the androgen receptor (AR) resulting in increased AR protein expression [100]. In liver cancers, templated insertion events also result in duplications and overexpression of *TERT* [1]. Apart from protein-coding genes, genes encoding ncRNAs can also contribute to the occurrence and development of cancer through CNA [105].

However, it is important to note that in many cases, amplification alone does not account for the observed increases in gene expression patterns owing to the influence of epigenetics [3].

### Fusion genes encoding novel oncogenic proteins

Approximately 82% of gene fusions are associated with structural variants [106]. Inversions or translocations may produce chimeric mRNAs encoding novel oncogenic proteins. Another known mechanism for gene or lncRNA activation by structural variations is the swapping of strong and weak promoters in the context of gene fusions [104]. Fusion mRNAs maintain their protein-coding sequences while being transcriptionally induced or repressed by swapping the 5′ ends (including the promoter). For example, anaplastic lymphoma kinase (*ALK*) rearrangement results in the *EML4-ALK* fusion oncogene, which is found in approximately 3–7% of all non-small-cell lung cancers (NSCLC) with distinct clinicopathological characteristics [107, 108]. Both classical chromothripsis and balanced chromothripsis can act as a source of fusion oncogenes *EML4-ALK* in NSCLC [29]. Chromoplexy can result in gene fusion (e.g., *EWSR1-ETS, BCLAF1-GRM1, SS18-SSX1,* and *FN1-FGFR1)* or gene amplification in multiple cancer types [109, 110].

Moreover, a nonprotein-encoding gene can be involved in fusion with other genes, resulting in the synthesis of an abnormal protein with alternative functions that participates in the cancer process [111, 112]. In medulloblastoma, chromothripsis results in a non-coding host gene *PVT1* (8q24.21) forming recurrent gene fusions including *PVT1-MYC* and *PVT1-NDRG1* [112]. The fusion gene *PVT1-MYC* and the positive feedback mechanism between them can be involved in the synergistic promotion of tumorigenesis [105].

### Alteration in epigenetics caused by structural variation interferes with gene expression

#### DNA methylation alterations result from the rearrangement of differentially methylated genomic regions or the altered expression of epigenetic factors in structural variation

DNA methylation changes can result from multiple mechanisms, such as alterations of epigenetic factors resulting from structural variants, genomic rearrangements, and DSB repair, thereby affecting the expression of some genes. First, DNA repair of DSBs results in alteration of CpG island methylation at the repair site, accompanied by corresponding changes in gene expression [98, 113]. Such structural variation-associated DNA methylation alterations involve the rearrangement of differentially methylated genomic regions [98]. Second, the disruption or overexpression of genes involved in methylation (e.g., *DNMTs,* and *NSD2*) is involved in DNA methylation alterations in structural variation. The overexpression of epigenetic factors resulting from structural variants is also associated with changes in the 3D chromosome structure [114, 115]. For example, overexpression of histone methyltransferase, *NSD2,* is induced in multiple myeloma (MM) with t(4;14), leading to changes in the 3D organization (including A/B compartmentalization and TADs) involving chromatin modifications such as the expansion of H3K36me2 [115]. Subsequently, expansion of H3K36me2 outside of active gene bodies increases chromatin accessibility to favor transcription factors and CTCF binding, thereby altering gene expression [115].

#### Alteration in TADs caused by structural variation results in inappropriate interactions between genes and regulatory elements

Bidirectional relationship between 2D chromatin and 3D genome organization has been implicated in gene regulation [115]. There has been increasing interest in the recognition of spatial genome organization as a vital factor in the formation of chromosomal rearrangements and carcinogenesis [62]. Changes in the TADs caused by structural variation also play a vital role in carcinogenesis by interfering with gene expression. Regulatory RNA can be recruited to specific genomic loci as scaffold-binding RNA-binding proteins (RBPs) such as CTCF to form specific protein complexes on chromatin [116, 117] (Fig. 2c). RNAs can bind and regulate CTCF and cohesin at chromatin boundaries and are required for the formation of chromatin loops through proper interaction with the RNA-binding region (RBRi) in CTCF [117–120]. Considering that loss of RBRi disrupts a subset of CTCF-mediated chromatin loops, CTCF loops were thought to have at least two classes, including RBRi-independent and RBRi-dependent loops [119].

Disruption of DNA integrity and structural variation often results in a change of TADs or chromatin architecture [121]. Approximately 5.0%, 8.5%, 12.8%, and 19.9% of all deletions, inversions, duplications, and complex events affect the boundaries of the TADs (specifically, spanning the entire length of a boundary), respectively, [122]. Deletions tend to occur within the same TAD, while duplications are often involved in regions across different TADs [122]. In MM and PCa, TADs are greater in number but smaller in size on average compared to normal cells [123, 124]. However, extensive changes in TAD size have little impact on gene expression [125]. The essential reason lies in the effect of inappropriate interactions between neighboring genes and regulatory elements on gene expression [126]. Super-enhancers tend to be preferentially insulated by strong boundaries to keep away from genes in adjacent TADs [38]. Enhancer-hijacking is considered a rare event and a mechanism exploited by genomic rearrangements [125, 127]. For many genes, structural variants are remarkably related to elevated numbers or greater proximity of enhancer regulatory elements near the gene [3, 127]. In medulloblastoma, enhancer-hijacking resulting from somatic structural variants has been demonstrated to activate the proto-oncogenes *GFI1* and *GFI1B* [128]. The rearrangement of two genomic regions with dramatically different methylation landscapes provides an explanation for structural variant-associated DNA methylation alterations [98].

**Fig. 3:**
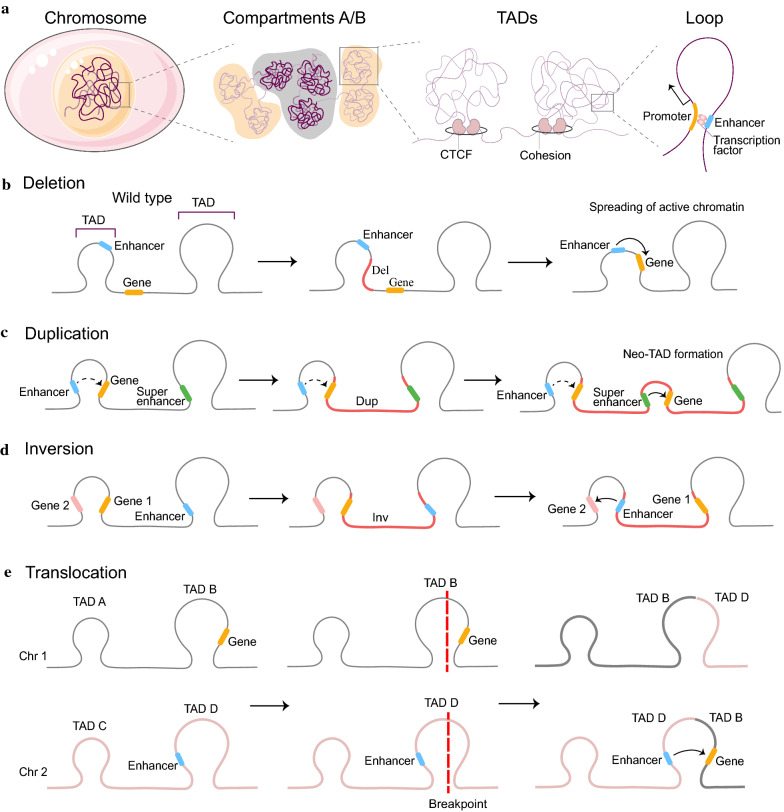
TAD and oncogene activation. **a** Hierarchical layers of chromatin organization. **b–e** Oncogene activation through different TAD rearrangements

Structural variation can result in the deletion or mutation of boundaries of TAD or insulated neighborhoods (INs) to produce a fusion of the two neighboring TADs or spreading of active chromatin, which establishes the promoter–enhancer interactions of an oncogene [5, 99]. In lung squamous carcinoma, deletion of the TAD boundary results in the spread of active chromatin and subsequent *IRS4* overexpression [129] (Fig. 3b). Similarly, in T-cell acute lymphoblastic leukemia (T-ALL), 113 recurrent deletions have been shown to overlap INs boundaries and result in activation of proto-oncogenes such as *TAL1* and *LMO2* in 6 affected INs [130]. In PCa, a deletion (17p13.1) bifurcates a TAD containing the *TP53* tumor suppressor gene into distinct smaller TADs that comprise the dysregulation of several genes [124]. At the same time, CNAs are associated with the formation of new domain boundaries in cancer cells [124]. However, it is important to note that a fusion of adjacent TADs caused by the removal of all major CTCF sites at the boundary and within the TAD does not exert major effects on gene expression owing to the existence of cohesin [126]. PCAWG analyses concluded that only 14% of the boundary deletions resulted in expression levels of nearby genes by over twofold [122].

The other mechanism is to produce neo-TADs by inversion or tandem duplications to form new regulatory interactions without directly affecting TAD boundaries [129, 131]. In AML with inv(3)/*RPN1-EVI1*, an inversion on chromosome 3 results in the fusion of two TADs. Therefore, the ectopic enhancer from *GATA2* TAD translocates to *EVI1* TAD, contributing to the activation of the *EVI1* oncogene with characteristics of a super-enhancer and decreased *GATA2* expression [132] (Fig. 3d). In rhabdomyosarcoma, a novel TAD encompassing the *PAX3-FOXO1* fusion resulting from t(2;13)(q35;q14) translocation, allows for interactions between the *PAX3* promoter and potential *FOXO1* enhancers [133]. In colorectal cancer, tandem duplications include the *IGF2* locus, TAD boundary, and a super-enhancer in adjacent TAD to mediate a de novo 3D contact domain between preexisting TADs, resulting in high-level overexpression of *IGF2* [129] (Fig. 3c)*.* In contrast, the expression levels and expression patterns of genes in the adjacent ‘parent TADs’ were not affected. Consistent with this, Gong et al*.* reported that super-enhancer elements and strong TAD boundaries are frequently co-duplicated in cancer patients [38] (Table 1).

**Table 1:** Selected examples of genes altered by structural variation in cancers

Malignancy	Structural variation type	Affected gene	Alteration in TADs	Effect	References
T-ALL	Deletion	*TAL1, LMO2, *etc.	Deletion of a loop boundary CTCF site	Key oncogenic drivers *TAL1* and *LMO2* expression from the silent state	[130]
Lung squamous carcinoma	Deletion	*IRS4*	Deletion of TAD boundary or insulator	*IRS4* overexpression caused by new enhancer–promoter interactions	[129]
Prostate cancer	Deletion (17p13.1)	*TP53*	Bifurcation of a single TAD into two distinct smaller TADs	Dysregulation of several genes	[124]
Colorectal cancer	Tandem duplications	*IGF2*	Formation of neo-TAD	De novo formation of a 3D contact domain comprising *IGF2* and a lineage-specific super-enhancer	[129]
Lung adenocarcinoma	Inv (2)(p21;p23)	*EML4-ALK*	–	–	[107]
AML	Inv(3)(q21;q26.2)	*RPN1-EVI1*	Fusion of two TADs	*EVI* oncogene expression caused by new enhancer–promoter interactions	[132]
Rhabdomyosarcoma	t(2;13)(q35;q14)	*PAX3-FOXO1*	Formation of neo-TAD		[133]
AML	t(8;21)	*RUNX1-ETO*	–	–	[78]

*AML* acute myeloid leukemia, *CTCF* CCCTC-binding factor, *TAD* topologically associated domains, *T-ALL* T-cell acute lymphoblastic leukemia

#### LncRNAs are involved in the alteration of TADs and gene regulation in structural variation

Remarkably, ncRNAs also play a role in the carcinogenesis mechanism of structural variation. A subgroup of positionally conserved lncRNAs, so-called topological anchor point (tap)RNAs, refers to those located at topological anchor points (chromatin loop anchor points and chromatin boundaries) and are misregulated in selected tumor types [134]. These tapRNAs are co-expressed with their neighboring genes in a tissue-specific manner and regulate the genes by affecting topological conformations [134] (Fig. 2d). For example, although in AML, overexpression of HOX genes, which have been identified as a dominant mechanism of leukemic transformation, has been attributed to specific chromosomal rearrangements [135]. A recent study demonstrated that the expression of lncRNA HOXA transcript at the distal tip (HOTTIP) mediates alteration of TADs of the AML genome to drive aberrant posterior HOXA gene expression and is thereby sufficient to initiate leukemic transformation of hematopoietic stem cells [135].

Apart from participating in the formation of structural variation, regulatory RNAs, especially lncRNAs, can assist in modulating target gene expression by facilitating chromatin remodeling, promoting enhancer–promoter interactions, or directly contributing to specific chromatin modification activities [116, 136, 137] (Fig. 2e, f). In breast cancer, overexpression of lncRNA RUNXOR alters the spatial chromatin structure of the *RUNX1* gene, the interaction between the promoters and enhancers, as well as methylation modifications of histones, to regulate the different transcripts of RUNX1 expression levels [137]. RNA has been shown to directly interact with multiple classic DNA-binding transcription factors and epigenome regulators such as components of the Polycomb repressive complex 2 (PRC2), including enhancer of zeste homolog 2 (EZH2) and SUZ12 [78, 138]. After recruiting PRC2 via EZH2 or other components to the targeted locus, some lncRNAs such as Kcnq1ot1 and Xist/RepA can promote the trimethylation of H3K27 in the targeted locus, thus silencing specific genes [139]. Chromatin-associated RNAs can also appear to form ‘RNA clouds’ over specific clusters of active gene promoters and their distal enhancers or another transcriptionally active TAD through long-range chromatin interactions and are vital for the formation of an active chromatin domain [140].

## Clinical value and application

### Detection of structural variants helps in early screening and subtype classification of tumors

Driver structural variants and point mutations often arise in the early decades of life, long before their clinical presentation [29, 103, 109, 141]. Therefore, the identification of structural variants at premalignant stages presents opportunities for the screening and identification of high-risk populations, and the subsequent monitoring and/or provision of early anticancer interventions. For example, chromosome 3p loss as the initiating driver occurs in childhood or adolescence, decades before clear cell renal cell carcinoma is diagnosed [103]. Compared to a constrained set of common drivers at the early stages of tumor development, a nearly fourfold diversification of driver genes and increased genomic instability are involved in late stages [141]. Such long latency of genetic aberrations offers early detection and long therapeutic windows before they reach their full malignancy potential [103]. Certain driver structural variants can be used for diagnostic purposes in specific cancers. For example, Carver et al*.* reported that *TMPRSS2-ERG* translocation appears to be an early event in PCa and may be related to the progression from high-grade prostatic intraepithelial neoplasia (HGPIN) to cancer [142]. However, it must be noted that no drivers were identified in approximately 5% of cases [4].

Until now, whole-exome sequencing has been used to identify genomic alterations in cancer patients. In the future, as the costs of next-generation sequencing and 3D genome technologies (such as Hi-C) decrease, paired samples from a patient’s cancerous and normal tissues can be sequenced to access all types of mutations as well as 3D genome disorganization in clinical oncology [2, 143]. The advent of single-cell Hi-C now allows for the detection of rare cells, as well as for the exploration of the heterogeneity of chromosomal conformation in cancer [144].

Moreover, as an important type of mutation, structural variants can play a significant role in cancer subtype classification [145]. In current clinical practice, cancer subtype classification based on driver mutations has been applied in some hematological cancers (e.g., myeloproliferative neoplasms, AML) and solid tumors (e.g., pancreatic cancer, breast cancer). The combination of histologic type, stage, and subtype classification can further improve stratification for intervention and prediction of therapeutic responses.

### Structural variants provide therapeutic targets and prognostic biomarkers of tumors

Identification of specific structural variations has aided the development of novel therapeutic targets and guided precision cancer treatment, which has achieved overwhelming success in improving survival rates in patients. In NSCLC, *ALK* rearrangements lead to oncogenic transformation through a constitutively active tyrosine kinase and downstream oncogenic signaling activation. This can be effectively targeted through the available ALK tyrosine kinase inhibitors (TKIs) such as crizotinib [146]. Moreover, 70% of *EML4-ALK* fusions disrupt tandem atypical propellers in the *EML* domain, leading to a structurally unstable fusion protein that relies on molecular chaperons to remain stable and thereby conveys sensitivity to Hsp90 inhibitors [147]. In addition, the HER-2 (also known as ERBB2) oncogene can be amplified through a series of structural variations in approximately 20% of all breast cancers [148]. HER-2 targeted therapeutic agents such as trastuzumab and pertuzumab have been applied for *HER2*-amplified breast cancers [148, 149]. In contrast, these structural variations result in gene inactivation and make it difficult to develop targeted therapeutics. Nonetheless, resistance remains a theme demanding the timely development of novel targeted therapeutics [146]. During subsequent stages of cancer progression, structural variation can further influence the accumulation of acquired resistance [150]. For example, different *EML4-ALK* variants in NSCLC patients impact the potential development of resistance mutations (especially G1202R) after TKI treatment [150, 151].

In addition, TAD formation and disruption, as well as changes in the chromosome organization during tumor evolution, may provide a novel perspective for elucidating additional mechanisms of structural variation and the development of therapeutic targets with improved efficacy. With the development of CRISPR/Cas9, the 3D structures of chromatin and the genome may be used in the development of novel therapeutic targets such as the CTCF boundary and ncRNAs [152].

In contrast, certain structural variations can be useful as biomarkers in cancer prognosis [109, 153]. Combining structural variation and clinical data can also increase prognostic accuracy. For example, Ewing sarcoma with chromoplexy rearrangement represents a more aggressive variant and it also portends a possible relapse and poor prognosis [109]. Chromothripsis is associated with shorter overall survival in patients with colorectal cancer [49].

## Future perspectives

Because of the ubiquitous prevalence and clinical importance of chromosome structural variations in cancer, we must explore and elucidate their underlying mechanisms. Advances in methods for mapping chromosome architecture and whole-genome sequencing have provided new insights into the complexity of cancer genome rearrangements. We might be able to define further rearrangement processes and thus unravel the causes of structural variation-driven human cancers. However, whether there are as yet undiscovered new types of chromosomal structural variations and mechanisms of formation or carcinogenesis remains to be confirmed.

Furthermore, what are the key driver components and mechanistic underpinnings of specific structural variation? It is clear from these studies that the 3D chromatin state plays a role in the mechanisms of formation and carcinogenesis, in structural variation. However, our understanding of key driver factors of structural variation remains insufficient and will necessitate future 3C and Hi-C-based studies to monitor dynamic structural variations. Further research is required to dissect the multiple roles of higher-order chromatin structure, ncRNAs, histone modifiers, and so on in key mechanistic steps in the process of structural variation. Development of single-cell sequencing and multi-omics assays may help provide more in-depth insights into cancer genomics and biology as well as clinical and therapeutic research.

## Data Availability

Not applicable.
